# Topical Application of Magnetized Saline Water Hydrogel Promotes Rapid Pressure Ulcer Healing in Hospitalized Elderly Patients: An Acute Care Case Series

**DOI:** 10.7759/cureus.87421

**Published:** 2025-07-07

**Authors:** Federico Ghidinelli, Gabriele Zanolini, Piercarlo Minoretti

**Affiliations:** 1 Geriatrics, Agenzia di Tutela della Salute, Brescia, ITA; 2 General Medicine, Azienda Socio-Sanitaria Territoriale della Franciacorta, Chiari, ITA; 3 Occupational Health, Studio Minoretti, Oggiono, ITA

**Keywords:** acute care, autophagy, elderly, hospitalization, magnetized water, pressure ulcers, wound care

## Abstract

Pressure ulcers, particularly among elderly patients with acute medical conditions, represent a significant clinical challenge and are associated with substantial morbidity and healthcare resource utilization. Autophagy, a fundamental cellular pathway involved in protein and organelle degradation, has emerged as a critical regulator of tissue repair and wound healing by orchestrating inflammatory cell activation, extracellular matrix deposition, and keratinocyte proliferation and differentiation. Topical application of magnetized saline water (MSW) may increase the expression of autophagy biomarkers in skin tissue, representing a potentially efficacious management strategy for wound care. We conducted a prospective observational case series to explore the clinical utility of a topical hydrogel containing 95% MSW in the management of stage 2‒3 pressure ulcers in elderly patients admitted to an acute medical unit. Five geriatric inpatients (age range: 75-93 years) who developed hospital-acquired pressure ulcers during acute care hospitalization were included in the study. Following standard wound cleaning protocols, the topical MSW hydrogel was applied daily to the affected areas. Wound healing progression was monitored via clinical photography, assessment of granulation tissue formation, and documentation of any adverse events. All patients exhibited effective wound healing, with clear clinical improvement observed as early as three days and up to 10 days following initiation of hydrogel application. Notable findings included granulation tissue formation, enhanced wound bed vascularization, progressive wound margin contraction, and active epithelial advancement. Intriguingly, these favorable outcomes were achieved despite the presence of severe comorbidities, including acute heart failure, pneumonia, and multi-organ dysfunction, which are typically associated with impaired wound healing in the elderly population. No adverse reactions related to the hydrogel application were evident. In summary, the findings from this case series suggest that an MSW-based hydrogel capable of stimulating autophagy may represent a promising therapeutic strategy for the management of pressure ulcers in acutely ill elderly patients. Further controlled studies are warranted to confirm these preliminary observations and to establish the role of MSW-based preparations within the broader context of wound care in geriatric medicine.

## Introduction

Hospital-acquired pressure ulcers represent a prevalent and significant clinical challenge among geriatric patients [1], with incidence rates ranging from 4% to 49% in critical care settings [2] and associated with a two-fold elevation in mortality risk relative to patients without pressure injuries [3]. Not surprisingly, traditional wound management strategies in acute care facilities commonly encounter inherent complexities, resulting in extended hospitalizations (57% increase) and elevated 30-day readmission rates (22% increase) [4]. Furthermore, the inherent operational constraints of acute care environments, including frequent patient repositioning for diagnostic procedures and therapeutic interventions, often compromise adherence to optimal wound management protocols [5]. This complex clinical milieu, characterized by the convergence of acute physiological stress, prolonged immobility, nutritional compromise, and inflammatory dysregulation, establishes multifaceted barriers to effective wound healing [6]. Concurrently, the systemic inflammatory response inherent to acute illness poses significant hurdles to normal tissue repair mechanisms, while commonly administered acute care medications, including antibiotics, non‐steroidal anti‐inflammatory drugs, corticosteroids, and anticoagulants, may further impair tissue regeneration processes [7].

Recent advances in understanding cellular repair mechanisms have identified autophagy as a master regulator of tissue homeostasis and regeneration [8]. This highly conserved process enables cells to survive stress by recycling damaged components and generating metabolic substrates [9]. In wound healing, autophagy coordinates inflammation resolution, promotes angiogenesis, and facilitates the phenotypic transitions required for tissue repair [10]. Notably, autophagic function becomes progressively dysregulated during aging [11] and acute inflammatory states [12], thereby contributing to compromised healing capacity in vulnerable elderly populations. In this context, non-pharmacological interventions targeting autophagy enhancement at pressure injury sites represent a potentially transformative approach for managing hospital-acquired pressure ulcers.

Magnetized water, an emerging technology with diverse applications spanning agriculture to medicine [13], results from magnetization processes that induce structural modifications in water molecules, including altered hydrogen bonding patterns, modified cluster formation, reduced surface tension, and enhanced electrical conductivity, which collectively contribute to its bioactive properties [14]. Notably, recent investigations have demonstrated that magnetized saline water (MSW) can significantly activate autophagy pathways in both in vitro [15,16] and clinical studies [17,18]. While topical application of MSW has been recently reported to promote autophagy-mediated healing of chronic wounds in elderly patients [19], the therapeutic potential of this approach within the challenging context of acute care settings remains underexplored. Here, we present a case series examining the clinical application of MSW hydrogel in managing pressure ulcers among acutely hospitalized elderly patients.

## Case presentation

Clinical setting and study patients

This prospective case series was conducted at a 45-bed general acute care unit at the tertiary referral center, "Mellino Mellini", Azienda Socio-Sanitaria Territoriale della Franciacorta, Chiari, Italy, between November 2024 and January 2025. The unit specializes in the management of complex geriatric conditions requiring intensive monitoring and multidisciplinary intervention.

Patient inclusion criteria comprised: (i) age ≥ 75 years, (ii) acute medical illness necessitating inpatient hospitalization, and (iii) development of hospital-acquired stage 2-3 pressure ulcers according to the National Pressure Ulcer Advisory Panel classification system [20]. Exclusion criteria encompassed: (i) clinical evidence of wound infection, (ii) requirement for surgical debridement, (iii) concurrent participation in other wound care studies, and (iv) inability to provide informed consent. Written informed consent was obtained from all participants or their legally authorized representatives. Patient confidentiality was maintained through coded identification systems.

The study product was a hydrogel containing 95% MSW (Gel Puro; Aquavis, Brescia, Italy), produced through magnetic field exposure of a multi-electrolyte solution consisting of 0.9% NaCl, 0.011% KCl, 0.009% CaCl_2_, 0.007% MgCl_2_, 0.007% ZnCl_2_, and 0.007% AlCl_3_, which underwent magnetization at 3000 Gauss for two hours. The standardized wound management protocol comprised: (i) gentle lesion irrigation using sterile water to remove debris and exudate, (ii) daily application of an adequate quantity of MSW hydrogel to ensure complete wound bed coverage, (iii) placement of a non-adherent primary dressing followed by sterile secondary gauze, and (iv) daily dressing changes executed by certified nursing personnel. Additional topical wound management interventions were not permitted during the study period. Daily follow-up assessments were performed by trained nursing staff, and digital photographs were obtained from all participants.

Case 1

**Figure 1:**
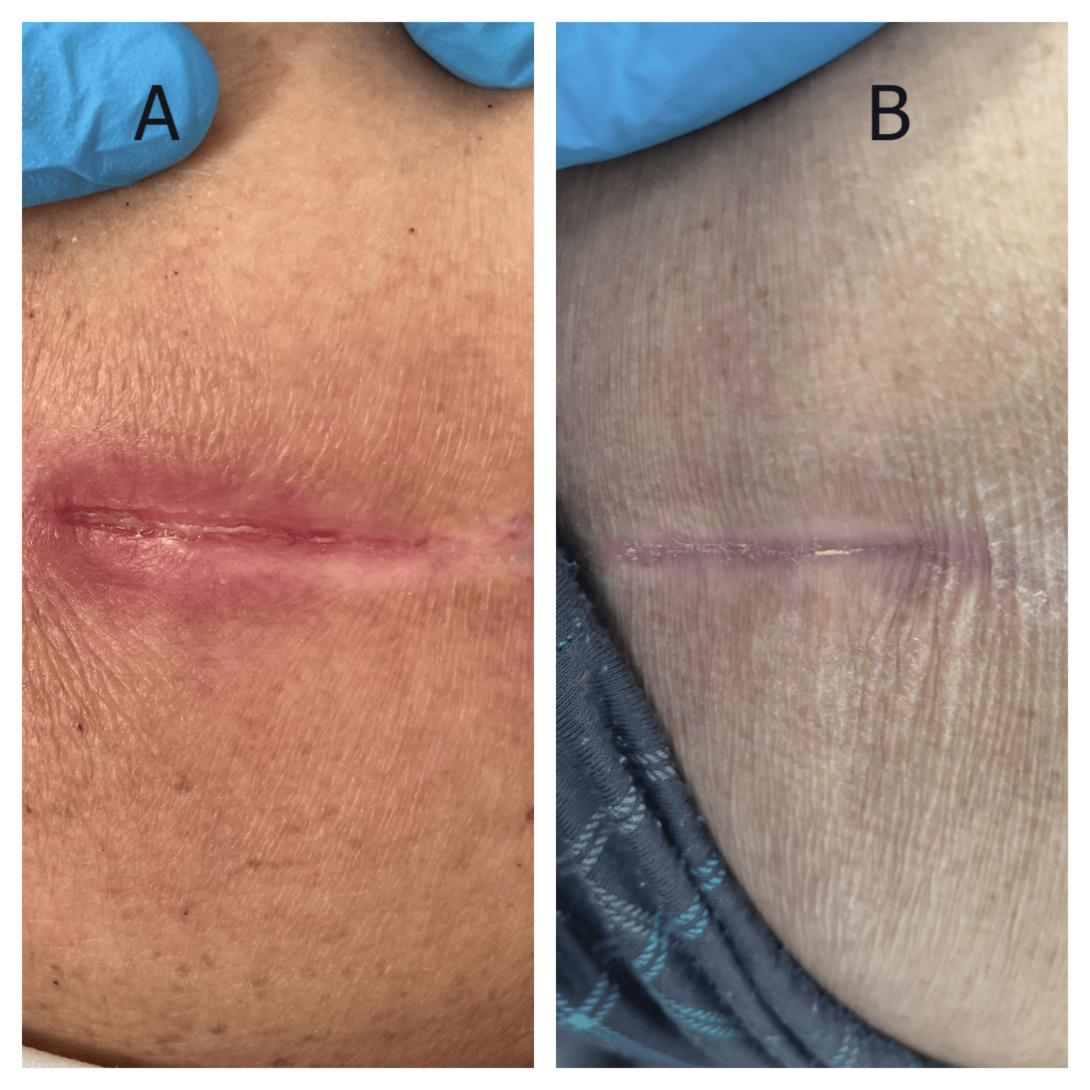
Response of a pressure-related linear wound to MSW hydrogel management in a patient with acute heart failure (Case 1) (A) Initial presentation showing a linear wound with partial-thickness tissue loss, exposed dermis, and early granulation tissue formation. (B) Day 10 following MSW hydrogel application, demonstrating complete wound closure with well-healed linear scar formation and resolution of surrounding tissue inflammation. MSW: magnetized saline water

A 75-year-old man was admitted with acute-on-chronic heart failure precipitated by atrial fibrillation with rapid ventricular response. His medical history included coronary artery disease with prior myocardial infarction, chronic obstructive pulmonary disease, and recently diagnosed anal carcinoma. Initial management required continuous cardiac monitoring, intravenous diuretics, and oxygen therapy. On hospital day 3, nursing assessment identified a linear pressure-related wound on the sacral area, attributed to prolonged positioning during acute stabilization. The wound presented as a linear defect measuring approximately 3.5 × 0.8 cm with partial-thickness tissue loss (Figure 1A). The wound edges were well-defined with visible pink granulation tissue at the base and minimal surrounding erythema. The perilesional skin demonstrated hyperpigmentation consistent with chronic pressure changes. MSW hydrogel management was initiated on hospital day 4. By day 7, progressive epithelialization was noted from the wound margins with contraction of the wound length. At day 10, the wound achieved complete closure with formation of a well-healed linear scar and significant improvement in surrounding skin quality (Figure 1B).

Case 2

**Figure 2:**
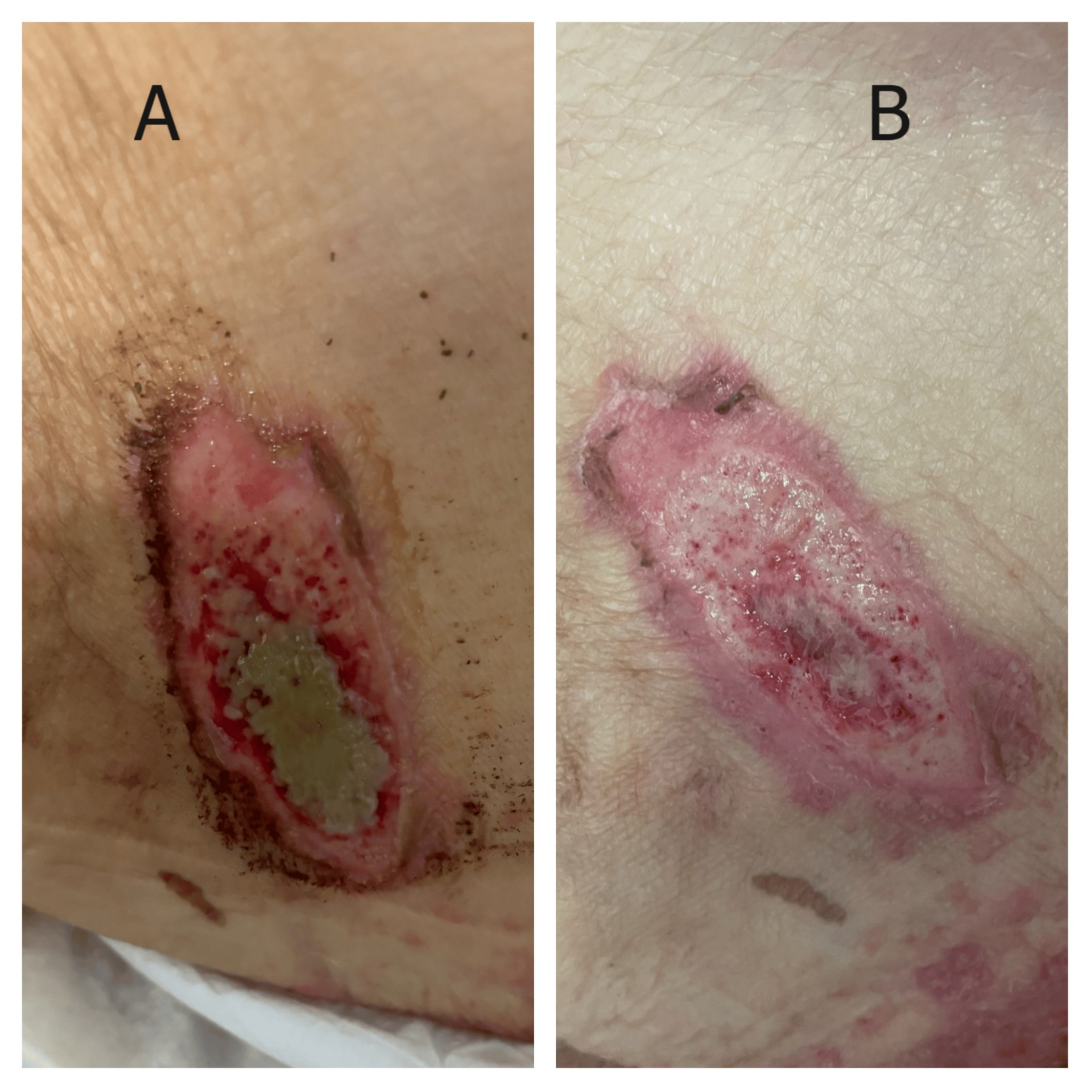
Response of a gluteal pressure ulcer to MSW hydrogel management in a critically ill patient (Case 2) (A) Initial presentation showing an elongated gluteal wound with extensive slough (60%), greenish-gray necrotic debris, and perilesional tissue maceration. (B) Day 7 post-debridement and MSW hydrogel application, demonstrating complete slough resolution, uniform pink granulation tissue formation, epithelializing wound edges, and significant wound contraction in a patient with cardiogenic shock and multiorgan dysfunction. MSW: magnetized saline water

An 84-year-old man presented with cardiogenic shock secondary to decompensated heart failure. Comorbidities included stage 4 chronic kidney disease, chronic atrial fibrillation, and peripheral arterial disease. Initial stabilization required mechanical ventilation, vasopressor support, and continuous renal replacement therapy. During the patient’s intensive care unit stay, a pressure ulcer developed over the right gluteal region. Initial assessment revealed an elongated wound measuring approximately 4.5 × 2.0 cm with full-thickness tissue loss extending through the dermis (Figure 2A). The wound bed contained approximately 60% slough with greenish-gray necrotic debris centrally and surrounding tissue maceration. Evidence of perilesional erythema and tissue breakdown was present. Following debridement, MSW hydrogel application commenced. Within 72 hours, the wound bed demonstrated marked improvement with healthy pink granulation tissue replacing the necrotic material. By day 7, the wound showed significant contraction with uniform granulation tissue filling the entire wound bed and well-defined epithelializing edges (Figure 2B). The transition from a wound with extensive slough to robust granulation tissue was notable given the patient’s critical illness and multiple organ dysfunction.

Case 3

**Figure 3:**
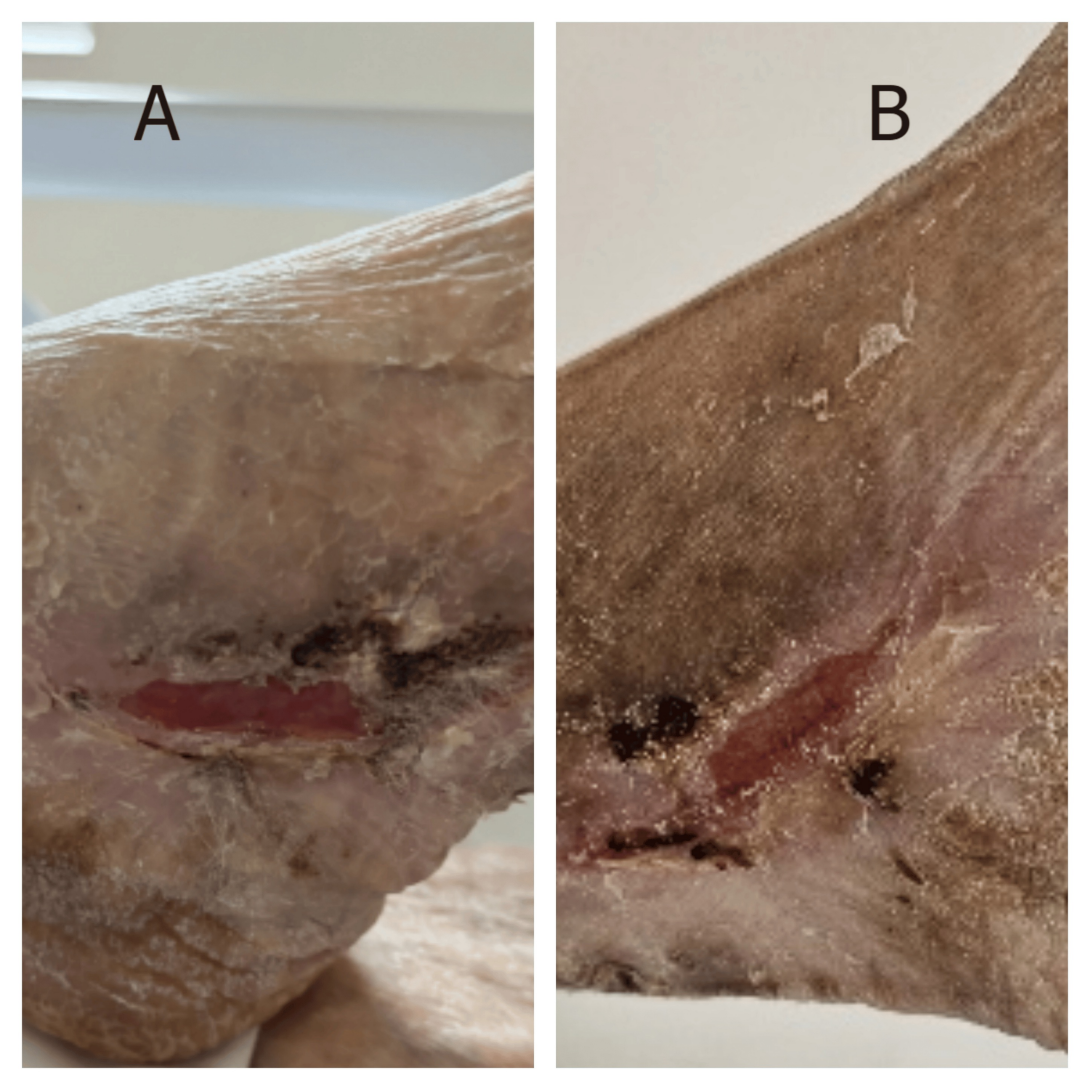
Response of a right calcaneal wound to MSW hydrogel in a diabetic patient with acute kidney injury (Case 3) (A) Admission finding showing a calcaneal wound with extensive surrounding hyperkeratosis and desquamating skin over entire heel. (B) Day 5 demonstrating wound contraction and mild softening of hyperkeratotic skin following MSW hydrogel application. MSW: magnetized saline water

An 80-year-old woman was admitted with severe dehydration secondary to viral gastroenteritis, complicated by acute kidney injury and hyperglycemic crisis. Her medical history included diabetes mellitus, stage 4 chronic kidney disease, and mild dementia. Initial management focused on fluid resuscitation, glycemic control, and electrolyte correction. Routine skin assessment on admission revealed a right calcaneal wound with extensive surrounding hyperkeratosis. The lesion measured approximately 2.5 × 0.7 cm and was surrounded by severely thickened, desquamating skin extending over the entire heel (Figure 3A). The extensive hyperkeratotic changes created a challenging wound environment with poor moisture retention and limited tissue flexibility. MSW management was initiated immediately upon admission. By day 5, significant improvement was evident with lesion size reduction and mild softening of the surrounding hyperkeratotic skin (Figure 3B). This response occurred despite ongoing metabolic derangements and an anatomically challenging location with inherently poor perfusion.

Case 4

**Figure 4:**
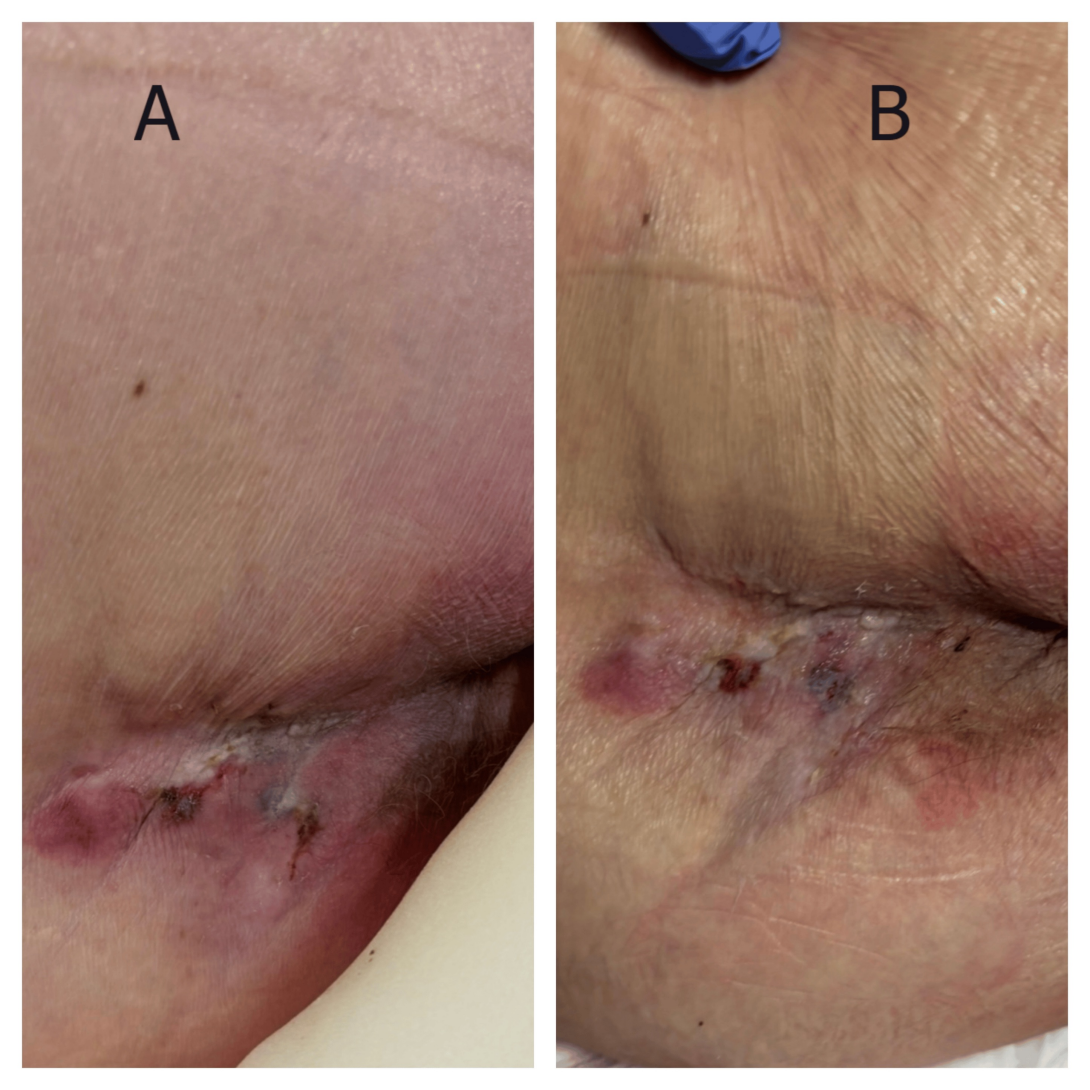
Response of multiple coalescing coccygeal pressure lesions to MSW hydrogel in a patient with bilateral pneumonia (Case 4) (A) Baseline appearance showing cluster of superficial to partial-thickness wounds with mixed tissue composition including slough, fibrinous exudate, and moisture-associated damage. (B) Day 3 demonstrating early healing response with increased granulation tissue, reduced slough, and improved periwound condition following MSW hydrogel application. MSW: magnetized saline water

A 93-year-old man presented with severe hypoxemia secondary to bilateral pneumonia. Significant comorbidities included dementia, chronic atrial fibrillation, and frailty syndrome. Initial treatment required high-flow oxygen therapy, broad-spectrum antibiotics, and intensive nursing care. Physical examination revealed multiple coalescing pressure lesions in the coccygeal region, presenting as a cluster of superficial to partial-thickness wounds (Figure 4A). The affected area measured approximately 5.0 × 4.0 cm overall, containing several discrete ulcerations with mixed tissue composition, including areas of slough, fibrinous exudate, and early granulation tissue. The wounds demonstrated features of combined pressure and moisture damage with irregular, macerated borders and varying depths. Surrounding skin showed erythema and moisture-associated changes. Given the patient's critical conditions and unfavorable prognosis, aggressive wound care was deemed inappropriate. Despite the abbreviated treatment period of only three days before care transition, MSW hydrogel application produced visible improvement (Figure 4B). The individual wounds showed signs of coalescence with cleaner wound beds, reduction in slough, increased pink granulation tissue formation, and decreased exudate. Periwound maceration improved notably. These changes occurred despite ongoing respiratory compromise and minimal nutritional intake.

Case 5

**Figure 5:**
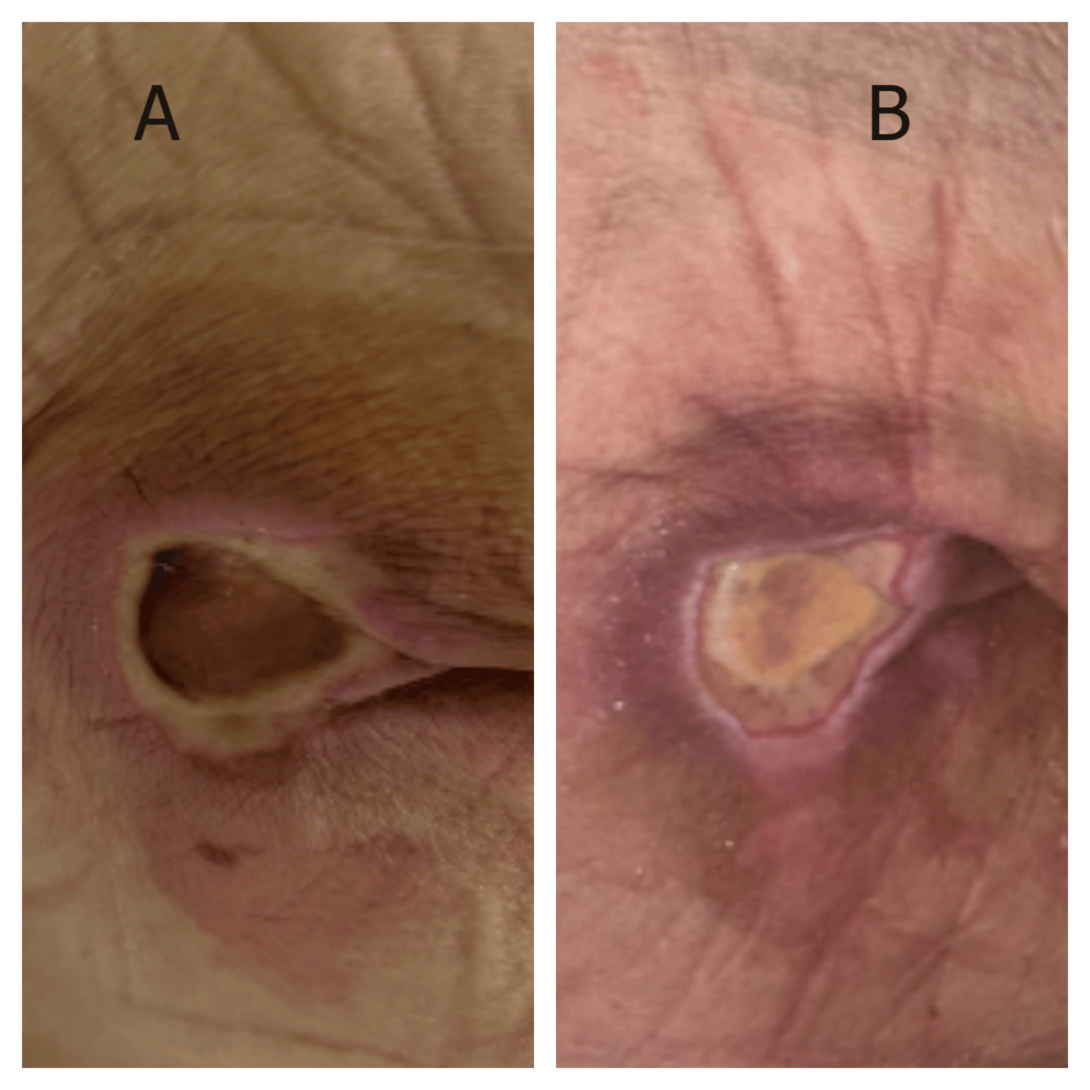
Response of a sacrococcygeal pressure ulcer to MSW hydrogel in a patient on maintenance hemodialysis with volume overload and pneumonia (Case 5) (A) Baseline showing extensive tissue loss with circumferential undermining. (B) Day 10 demonstrating significant tissue regeneration and wound contraction following MSW hydrogel application. MSW: magnetized saline water

An 85-year-old man on maintenance hemodialysis was admitted with volume overload and pneumonia. His extensive comorbidity profile included ischemic cardiomyopathy, type 2 diabetes with retinopathy, and severe peripheral vascular disease. The combination of uremia, diabetes, and vascular insufficiency created an extremely challenging wound-healing environment. Nursing assessment identified a sacrococcygeal pressure ulcer measuring approximately 3.0 × 2.5 cm with significant depth, creating a cavity-like defect (Figure 5A). The surrounding tissue showed signs of moisture damage and hyperpigmentation. The challenging anatomical location created additional moisture retention issues. MSW hydrogel management was initiated following gentle mechanical debridement of the lesion. By day 5, healthy granulation tissue began filling the cavity from the base. On day 10, the wound showed significant improvement with the cavity nearly completely filled with robust yellow-pink granulation tissue (Figure 5B). The wound opening contracted significantly, and the surrounding skin demonstrated marked improvement with reduction of maceration and improved tissue quality.

## Discussion

This case series documented remarkable wound healing responses to MSW hydrogel application in five acutely ill hospitalized elderly patients. Intriguingly, the observed effects, including granulation tissue formation, significant wound contraction, and reepithelialization, occurred despite clinical circumstances typically associated with profoundly impaired healing. Collectively, our findings demonstrate that, in acute care settings, MSW-based hydrogels may offer a promising therapeutic approach for the management of pressure ulcers, possibly through the activation of autophagy [19].

Systemic inflammatory responses characteristic of acute illness are known to suppress normal repair mechanisms through dysregulation of cytokine networks and impaired cellular metabolism [21]. Our patients exemplified these challenges, presenting with life-threatening conditions including cardiogenic shock, respiratory failure, and multiorgan dysfunction. While wound healing would be expected to stall or deteriorate under such circumstances, we observed that the topical application of an MSW hydrogel appeared to accelerate healing trajectories. The mechanism underlying the wound-healing effects of MSW gel appears to be primarily mediated by autophagy activation [15-19]. Intriguingly, the rapid onset of healing (often within 72 hours of initiation) suggests direct potentiation of intrinsic cellular repair machinery.

Our observations are in accordance with the emerging understanding of autophagy's role in tissue repair. Autophagy coordinates multiple aspects of wound healing including: (i) removal of damaged proteins and organelles that accumulate during cellular stress [22], (ii) generation of metabolic substrates through recycling [23], (iii) modulation of inflammatory responses [12], (iv) promotion of endothelial cells angiogenesis [24], and (v) facilitation of cellular differentiation required for tissue maturation [8]. In acute illness, where these processes become dysregulated, external autophagy activation may restore normal healing capacity. Several aspects of our findings warrant emphasis. First, the favorable responses occurred across diverse wound locations and stages, suggesting broad applicability. Second, healing progressed despite ongoing acute illness and minimal optimization of systemic factors. Third, the treatment protocol’s simplicity, consisting of once-daily application without complex wound preparation, makes it feasible in busy acute care settings. Fourth, no adverse events were observed despite the vulnerability of our patient population. We posit that the clinical implications of our findings may be substantial. Hospital-acquired pressure ulcers impose a considerable burden on healthcare systems, markedly prolonging hospital stays and exerting a significant negative impact on patient morbidity and mortality [1-3]. Current prevention and treatment strategies for geriatric patients in acute care settings exhibit limited effectiveness, highlighting the need for innovative management approaches [4,6]. Our results suggest that a management strategy capable of accelerating wound healing despite adverse systemic conditions has the potential to substantially transform current wound care paradigms. Consequently, the prospect of reduced complications, shortened hospitalizations, and enhanced patient quality of life merits further rigorous investigation.

Nevertheless, we acknowledge several limitations inherent to this preliminary study. Its observational design, small sample size, and absence of a control group constrain the strength of the conclusions. The heterogeneity of the patient population and variability in follow-up periods further complicate standardized outcome assessment. Moreover, the absence of tissue biopsies to confirm autophagy activation limits mechanistic validation. These limitations are somewhat offset by the real-world, acute care context and the inclusion of critically ill elderly patients who are often excluded from conventional wound healing studies. We believe that future research should address several critical questions. Randomized controlled trials directly comparing MSW hydrogels to standard wound care are essential to establish efficacy. Molecular studies incorporating tissue analysis for autophagy markers would provide mechanistic confirmation. Further investigation is needed to define optimal treatment protocols with respect to frequency, duration, and patient selection criteria. Long-term outcomes, including recurrence rates and quality of life metrics, should be systematically assessed. Finally, economic analyses comparing the costs and outcomes of MSW therapy to those of current therapies are warranted to inform healthcare policy and resource allocation.

## Conclusions

This case series demonstrates that topical application of an MSW hydrogel can facilitate rapid healing of pressure ulcers in acutely hospitalized elderly patients, likely mediated through autophagy activation. The observed wound healing responses, often within days, even in the context of critical illness and significant comorbidity burden, suggest that this novel intervention may effectively counteract the healing impairments commonly encountered in acute care environments. These preliminary findings underscore the urgent need for controlled clinical trials to rigorously assess the efficacy of MSW and its potential to redefine pressure ulcer management for high-risk, vulnerable patient populations.
